# 17 Exploring the feasibility of an mHealth application to enhance physical activity in community-dwelling older adults

**DOI:** 10.1093/eurpub/ckae114.065

**Published:** 2024-09-26

**Authors:** Kim Daniels, Jolien Robijns, Sharona Vonck, Annemie Spooren, Dominique Hansen, Bruno Bonnechère

**Affiliations:** Centre of Expertise in Innovation in Care, Pxl University College Of Applied Sciences and Arts, Hasselt, Belgium; Rehabilitation Research Center, Hasselt University, Diepenbeek, Belgium; Centre of Expertise in Innovation in Care, Pxl University College Of Applied Sciences and Arts, Hasselt, Belgium; Centre of Expertise in Innovation in Care, Pxl University College Of Applied Sciences and Arts, Hasselt, Belgium; Centre of Expertise in Innovation in Care, Pxl University College Of Applied Sciences and Arts, Hasselt, Belgium; Rehabilitation Research Center, Hasselt University, Diepenbeek, Belgium; Rehabilitation Research Center, Hasselt University, Diepenbeek, Belgium; Centre of Expertise in Innovation in Care, Pxl University College Of Applied Sciences and Arts, Hasselt, Belgium; Rehabilitation Research Center, Hasselt University, Diepenbeek, Belgium; Data Sciences Institute, Hasselt University, Diepenbeek, Belgium

## Abstract

**Background:**

Advancements in smartphone technology have paved the way for innovative interventions aimed at promoting physical activity (PA) and have led to the development of an mHealth app called MIA. The primary objective of this feasibility study is to comprehensively investigate the potential of the MIA app in effectively promoting PA and fostering sustained, healthy behaviors among its users.

**Methods:**

A diverse sample of 30 participants aged 65 or older, free of severe illness from the community was recruited. Baseline data was collected on demographics, assessed PA (IPAQ SF), measured PA enjoyment (PACES), and evaluated digital health readiness (DHRQ). Participants used the app while providing real-time feedback via a “think-aloud” protocol. A five-week usage period followed, with post-period PACES assessment and usability measures (SUS, UEQ, NPS, CSAT). We analyzed the app usage patterns with Power BI and gathered qualitative data through five focus groups. Both qualitative and quantitative analyses were employed, assessing clinical relevance alongside statistical significance, and descriptive statistics to summarize results were used.

**Results:**

The study included 30 participants, averaging 69.9±5.3 years old, with 17 females (56.7%) and 13 males (43.3%), primarily having completed higher education (50%) and displaying strong digital literacy skills (average 59.6±8.8). Regarding PA, 10% were inactive, 40% minimally active, and 50% vigorously active, indicating a highly active sample. Usability and acceptance were positive, with an SUS score of 77.41, CSAT score of 86.6%, and 87% expressing satisfaction. Power BI data showed participants engaged in an average of three workouts per week and read one to two articles weekly.

Focus groups supported quantitative findings, suggesting improvements like more motivational tips, app usage tutorials, clearer instructions for workout videos, expanded community activities, a reader tracker, enriched learning modules, and enhanced progression tracking.

**Discussion and conclusion:**

The Mia app shows feasibility in usage, acceptability, demand, and practicality, prompting further impact assessments. Positive feedback highlights the need for user-tailored improvements before clinical trials. However, limitations include a non-representative, digitally literate sample, potential bias from self-reported PA measures, and lack of blinding. Future research should explore less tech-savvy older adults’ perceptions, gauging accessibility and appeal to a broader audience.

